# The significance of coronary artery calcium score as a predictor of coronary artery stenosis in individuals referred for CT angiography

**DOI:** 10.34172/jcvtr.2020.34

**Published:** 2020-09-03

**Authors:** Reza Kiani, Hamidreza Pouraliakbar, Mohammad Javad Alemzadeh-Ansari, Ali Khademi, Mohamad Mehdi Peighambari, Bahram Mohebbi, Ata Firouzi, Ali Zahedmehr, Farshad Shakerian, Zahra Hosseini, Alireza Rashidinejad

**Affiliations:** ^1^Cardiovascular Intervention Research Center, Rajaie Cardiovascular Medical and Research Center, Iran University of Medical Sciences, Tehran, Iran; ^2^Department of Radiology, Rajaie Cardiovascular Medical and Research Center, Iran University of Medical Sciences, Tehran, Iran; ^3^Cardio-Oncology Research Center, Rajaie Cardiovascular Medical and Research Center, Tehran, Iran

**Keywords:** Coronary Artery Disease, Angiography, Coronary Calcium Score

## Abstract

***Introduction:*** Cardiovascular diseases, including coronary artery disease (CAD), are among the most common causes of death in the elderly population. Recent studies have found that coronary artery calcium score (CACS) is a strong independent predictor of CAD. Here we aimed to investigate the association between CACS and demographic, clinical, laboratory, and CT angiographic findings inpatients with suspected CAD.

***Methods:*** From June 2008 to August 2018, we retrospectively reviewed 219 consecutive patients suspected with CAD who were referred for CT angiography in Rajaie Cardiovascular, Medical, and Research Center. Medical records were reviewed, and relevant demographic, clinical, laboratory and imaging were collected.

***Results:*** A total of 219 patients with an average age of 62.64±12.39 were included. Twelve patients(5.5%) had normal coronary angiography, and 50.2% had mild CAD. An obstructive CAD was found in97 patients (44.3%). The median CACS was 76.4 (IQR, 13.0-289.1). The frequency of obstructive CAD was 28.1% in the CACS <100 group, and 67.0% in CACS >100 group (*P* < 0.001). On multiple logistic regression analysis, age (OR=1.04 [1.01-1.07], *P* = 0.006), CACS (OR= 4.31 [2.33-7.98], *P* < 0.001), and neutrophil to lymphocyte ratio (NLR) (OR = 0.82 [0.68-0.98], *P* = 0.027) were independent predictors of obstructive CAD.

***Conclusion:*** We found a direct association between higher CACS and obstructive patterns in coronary CT angiography. Our findings indicate that the possibility of the presence of obstructive CAD was higher among symptomatic patients with older age, lower NLR, and CACS >100.

## Introduction

Cardiovascular diseases, including coronary artery disease (CAD), are among the most common causes of death in the elderly population. According to the World Health Organization (WHO), cardiovascular diseases take nearly 18 million lives annually.^1^ The lifetime risk of developing CAD is estimated to be 49 percent in men and 32 percent in women.^2^ Therefore, identifying people at risk and early diagnosis is important.

Older age, male gender, hypertension, diabetes, dyslipidemia, obesity, smoking, and low physical activity are among the most established risk factors for cardiovascular diseases.^3,4^ Recent studies have found that coronary artery calcium (CAC) is a strong independent predictor of CAD.^5-8^ Calcification of the coronary arteries has an important role in the pathophysiology of atherosclerosis. CAC can be easily measured by noninvasive imaging methods, including electron-beam tomography (EBT) or multidetector computed tomography (CT).^9^ CAC score (CACS) measured by noncontrast cardiac CT scan is a low-radiation and relatively cheap test that provide a quantitative assessment of the overall coronary atherosclerotic burden.^10^ Growing evidence suggests that CACS is a useful test for risk stratification of both symptomatic and asymptomatic individuals.^11-13^ Higher CACSs has been shown to be associated with a higher risk of major cardiovascular events and all-cause mortality.^5,14,15^

Several studies have claimed that there are some associations between CACS and cardiac risk factors.^16-18^ However, there were great inconsistencies between the reported results. Here we aimed to investigate the association between CACS and demographic, clinical, laboratory, and CT angiographic findings in patients with suspected CAD. We also evaluated the predictive value of CACS beyond traditional cardiovascular risk factors for obstructive CAD.

## Materials and Methods

From June 2008 to August 2018, we retrospectively reviewed 219 consecutive patients suspected with CAD who were referred for CT angiography in Rajaie Cardiovascular, Medical, and Research Center, affiliated to Iran University of Medical Sciences. Inclusion criteria included all adult patients with stable angina candidate for CT angiography. Patients with a prior history of CVDs, chronic kidney disease, significant liver dysfunction were excluded. Also, who had history of previous percutaneous coronary intervention or coronary artery bypass grafting were excluded. The patient with suspicious or confirmed acute coronary syndrome excluded. Patients’ demographic, clinical, and laboratory findings were obtained from data bank.

### Computed tomography scanning protocol

Coronary CT angiography was performed with 192-slice CT scanner (SOMATOM FORCE, Forchheim, Germany). In all patients, a non-contrast enhanced scan (120-kV tube voltage and 3-mm slice thickness) to calculate the total CACS was performed prior to CCTA (120-kV tube voltage, 0.75-mm slice thickness, 0.3 or 0.4-mm reconstruction increment). A 50 -60-ml contrast (IOHEXOLE: Omnipaque 350 mgI/mL, GE HealthCare Inc, USA), followed by a 20-ml saline solution chaser, was injected at 3.5–5.5 mL/s. Nitroglycerine sublingually was administered immediately before contrast injection. The Agatston scoring method was used to measure the CACS.^19^ The total CACS was categorized as low (0–100) and high (>100). Coronary CT angiography scans were evaluated by experienced radiologists, blinded to the CACS results. Obstructive CAD was defined as [50% luminal narrowing of C1 coronary segment on CCTA.

### Statistical analysis

Statistical analyses were performed using the Statistical Package of Social Science version 25. Continuous variables are expressed as mean ± standard deviation (SD) and categorical variables are presented as absolute numbers and percentages. The independent samples *t* test or chi-square test was used to compare variables between the groups. Multivariate logistic regression analysis was performed to identify independent risk factors of obstructive CAD. A two-sided *P* < 0.05 was considered statistically significant.

## Results

A total of 219 patients were studied. Patients’ demographic, clinical, and laboratory findings are summarized in Table 1 and Table 2. The mean EF was 44.39±12.48. Twelve patients (5.5%) had normal coronary angiography, and nearly half of the participants had mild CAD. An obstructive CAD was found in 97 patients (44.3%). Other important CT angiographic findings were positive remodeling in nine patients (4.1%), napkin ring sign in four patients and low-density plaque in three patients. The median CACS was 76.4 (IQR, 13.0-289.1).

**Table 1:** Patients’ characteristics

**Characteristics**	
Age, years [mean±SD]	62.6±12.4
Male, n (%)	121 (55.3)
Risk factors, n (%)	
Diabetes	60 (27.4)
Dyslipidemia	93 (42.5)
Hypertension	120 (54.8)
Smoking^*^	51 (65.4)
Positive family history	44 (20.1)
Chief complaint, n (%)	
Chest pain	132 (60.3)
Dyspnea	105 (47.9)
SBP, mm Hg [mean±SD]	127.7±17.9
DBP, mm Hg [mean±SD]	78.2±12.4
Ejection fraction [mean±SD]	44.4±12.5
CCTA, n (%)	
Normal	12 (5.5)
Mild CAD	110 (50.2)
1-vessel disease	44 (20.1)
2-vessel disease	28 (12.8)
3-vessel disease	25 (11.4)
CACS, n (%)	
0-100	128 (58.4)
100-300	39 (17.8)
>300	52 (23.7)

CACS, coronary artery calcium score; CAD, coronary artery disease; CCTA, coronary computed tomographic angiography; SD, standard deviation * Data were available from 78 patients.

**Table 2:** Laboratory findings

Test	
Hemoglobin (g/dL)	13.4±1.8
White blood cells (/mm^3^)	7403.2±2571.0
Neutrophil (%)	65.3±11.3
Lymphocyte (%)	24.6±10.6
Platelets (/mm^3^)	193.2±55.0
Platelet-to-lymphocyte ratio	134.3±74.0
Neutrophil-to-lymphocyte ratio	3.5±2.2
Triglyceride (mg/dL)	144.6±86.2
Cholesterol (mg/dL)	153.4±40.5
LDL (mg/dL)	88.8±31.0
HDL (mg/dL)	39.7±10.1
Fasting blood glucose (mg/dL)	120.1±40.9
BUN (mg/dL)	21.3±15.6
Creatinine (mg/d)	1.1±0.6
ESR	18.2±14.5
HsCRP^*^(mg/L)	11.0±17.9

BUN: blood urea nitrogen; HsCRP: high-sensitivity C-reactive protein; ESR: erythrocyte sedimentation rate; HDL: high-density lipoprotein; LDL: low-density lipoprotein. *Data were available from 65 patients.

Patients’ characteristics are compared across the CACS groups in Table 3. Patients with higher CACSs were significantly older than patients with CACS<100 (*P <* 0.001). The frequency of obstructive CAD was 28.1% in the CACS< 100 group, and 67.0% in CACS>100 group (*P <* 0.001).

**Table 3:** Clinical characteristics according to CACS groups

	**CACS <100 (n=128)**	**CACS >100 (n=91)**	***P***
Age, years [mean±SD]	60.1±12.6	66.2±11.3	**<0.001**
Male, n (%)	65 (50.8)	56 (61.5)	0.130
Diabetes, n (%)	34 (26.6)	26 (28.6)	0.760
Dyslipidemia, n (%)	52 (40.6)	41 (45.1)	0.579
Hypertension, n (%)	70 (54.7)	50 (54.9)	1.000
Smoking, n (%)	33 (67.3)	18 (62.1)	0.806
Positive family history, n (%)	29 (22.7)	15 (16.5)	0.306
Chief complaint, n (%)			
Chest pain	76 (59.4)	65 (61.5)	0.781
Dyspnea	61 (47.7)	44 (48.4)	1.000
LVEF [mean±SD]	44.2±13.3	44.6±11.1	0.815
Obstructive CAD	36 (28.1)	61 (67.0)	**<0.001**
Remodeling	8 (6.3)	1 (1.1)	0.084
Hemoglobin (g/dL)	13.5±1.9	13.3±1.8	0.655
WBC (/mm^3^)	7594.1±2639.7	7134.5±2460.4	0.193
Neutrophil (%)	66.5±11.8	63.7±10.4	0.070
Lymphocyte (%)	24.2±10.6	25.2±10.6	0.511
Platelets(/mm^3^)	197.3±60.3	187.4±46.1	0.189
PLR	134.1±71.0	134.5±78.5	0.970
NLR	3.6±2.2	3.3±2.2	0.355
Triglyceride (mg/dL)	145.5±88.3	143.5±83.8	0.871
Cholesterol (mg/dL)	150.6±38.1	157.3±43.5	0.235
LDL (mg/dL)	88.0±29.2	89.8±33.4	0.668
HDL (mg/dL)	38.9±10.3	40.8±9.8	0.169
FBS (mg/dL)	120.2±43.8	119.8±32.9	0.954
BUN (mg/dL)	20.4±11.3	22.4±19.8	0.397
Creatinine (mg/d)	1.1±0.7	1.0±0.3	0.492
ESR	17.8±15.0	18.8±13.9	0.622
HsCRP(mg/L)	8.4±7.4	9.4±8.4	0.618

ESR, erythrocyte sedimentation rate; BUN, blood urea nitrogen; HsCRP, high sensitivity C reactive protein; FBS, fasting blood sugar; HDL, high density lipoprotein; LDL, low density lipoprotein; LVEF, left ventricular ejection fraction; NLR, neutrophil to lymphocyte ratio; PLR, platelet to lymphocyte ratio; WBC, white blood cells.

We also compared the characteristics of patients with and without obstructive CAD (Table 4). Patients with obstructive CAD were significantly older than patients with normal angiography or mild CAD (*P =* 0.001). Patients with obstructive CAD had significantly lower neutrophil to lymphocyte ratio (NLR) values compared to the other group (*P =* 0.005).

**Table 4:** Clinical characteristics according to stenosis status

	**Obstructive** **CAD + (n=97)**	**Obstructive** **CAD - (n=122)**	***P***
Age, years [mean±SD]	65.7±11.4	60.2±12.6	**0.001**
Male, n (%)	63 (51.6)	58 (59.8)	0.274
Diabetes, n (%)	37 (30.3)	23 (23.7)	0.290
Dyslipidemia, n (%)	50 (41.0)	43 (44.3)	0.680
Hypertension, n (%)	66 (54.1)	54 (55.7)	0.891
Smoking, n (%)	30 (63.8)	21 (67.7)	0.810
Positive family history, n (%)	28 (23.0)	16 (16.5)	0.308
Chief complaint, n (%)			
Chest pain	70 (57.4)	62 (63.9)	0.335
Dyspnea	60 (49.2)	45 (46.4)	0.686
LVEF [mean±SD]	44.8±11.7	44.0±13.1	0.649
Hemoglobin (g/dL)	13.3±1.8	13.5±1.8	0.467
WBC (/mm^3^)	7207.5±2548.4	7558.7±2588.7	0.316
Neutrophil (%)	63.8±9.6	66.6±12.4	0.074
Lymphocyte (%)	26.0±9.1	23.5±11.5	0.093
Platelets (/mm^3^)	195.0±50.9	191.7±58.2	0.658
PLR	124.4±57.2	142.1±84.5	0.066
NLR	3.0±1.8	3.8±2.4	**0.005**
Triglyceride (mg/dL)	139.5±72.9	148.7±95.6	0.433
Cholesterol (mg/dL)	153.6±40.3	153.3±40.8	0.945
LDL (mg/dL)	88.0±30.6	89.3±31.4	0.754
HDL (mg/dL)	39.5±9.3	39.9±10.7	0.806
FBS (mg/dL)	114.4±29.0	124.3±47.6	0.162
BUN (mg/dL)	20.5±8.1	21.8±19.4	0.574
Creatinine (mg/d)	1.1±0.9	1.0±0.2	0.180
ESR	19.2±14.8	17.5±14.3	0.398
HsCRP(mg/L)	10.1±8.6	7.7±6.9	0.221

ESR, erythrocyte sedimentation rate; BUN, blood urea nitrogen; HsCRP, high sensitivity C reactive protein; FBS, fasting blood sugar; HDL, high density lipoprotein; LDL, low density lipoprotein; LVEF, left ventricular ejection fraction; NLR, neutrophil to lymphocyte ratio; PLR, platelet to lymphocyte ratio; WBC, white blood cells.

On multiple logistic regression analysis, age, CACS, and NLR were independent predictors of obstructive CAD (Table 5). Older age and lower NLR values were associated with a higher probability of obstructive CAD. Patients with CACS>100 had a 4.31-fold greater risk of obstructive CAD.

**Table 5:** Independent predictors of obstructive CAD

	**OR (95% CI)**	***P***
Age	1.04 (1.01-1.07)	**0.006**
Male sex	0.75 (0.41-1-40)	0.371
CACS>100	4.31 (2.33-7.98)	**<0.001**
NLR	0.82 (0.68-0.98)	**0.027**
PLR	1.00 (0.99-1.00)	0.678

CACS, coronary artery calcium score; NLR, neutrophil to lymphocyte ratio; PLR, platelet to lymphocyte ratio.

## Discussion

In the present study, we evaluated the association between CACS and demographic, clinical, laboratory, and CT angiographic findings of patients suspected with CAD. In the univariate analyses, we found that patients with greater CACSs (>100) were older and the frequency of obstructive CAD was higher among them. When we compared the characteristics of patients with and without obstructive CAD in CT angiography, we realized that patients with obstructive CAD were older and had lower NLR values. Finally, we found that older age, lower NLR, and CACS>100 are independent significant predictors of the presence of obstructive CAD in CT angiography.

Previous studies have shown that increased CACS is significantly associated with increased cardiovascular risk factors and more severe coronary artery stenosis.^20-22^ Ho et al,^20^ reported that patients with higher CACS were older and the prevalence of male gender, hypertension, and significant CT angiographic stenosis were higher among them. Likewise, Ueda et al,^22^ noticed that greater CACSs in patients with suspected CAD was significantly associated with older age, male gender, and presence of hypertension, diabetes, and hypercholesterolemia. The also realized that the prevalence of obstructive CAD increased with the CACS. Similar to these studies, we found that the prevalence of obstructive CAD was greater in patients with CACS>100 compared with those with lower CAC levels (67.0% vs 28.1%). CACS>100 was associated with a 4.31-fold increased risk of obstructive CAD.

Older age was another independent risk factor for obstructive CAD in this study. We also found that the mean age of patients with increased CACS was significantly higher than patients with lower CACSs. This finding is in line with earlier studies which reported a direct association between age and CAC.^23^ Unlike the abovementioned studies, we did not find any significant association between CACS and male gender, diabetes, dyslipidemia, hypertension, and smoking.^20-22^ Variation in study design, CACS cut-off points, sample size, and analytic approaches could explain these inconsistencies.

Inflammation plays a pivotal role in the pathophysiology of atherosclerosis.^24^ Some earlier studies have shown that higher levels of inflammatory markers, including erythrocyte sedimentation rate (ESR), C-reactive protein (CRP), and inflammatory cytokines such as interleukin 1 (IL-1), IL-6, IL-10, monocyte chemoattractant protein-1 (MCP-1) and tumor necrosis factor-alpha (TNF-α) may predict poor cardiovascular prognosis.^24-26^ In the present study, we found no significant association between ESR or CRP and CACS. Also, we realized that none of these two inflammatory markers contributed to the risk of obstructive CAD. In line with our findings, two previous studies with relatively large sample sizes did not report strong associations between CRP and CACS.^27-29^ Lack of association between CACS and inflammatory markers in these studies may indicate that inflammation plays a non-significant role in calcification of coronary arteries.

Neutrophils secrete inflammatory mediators that promote plaque formation.^24^ Elevated NLR values were linked to adverse cardiovascular outcomes and higher mortality rates.^30^ Two Korean studies conducted in asymptomatic adults found a direct association between NLR and CACS.^31,32^ In contrast to these studies, we found lower levels of NLR among patients with higher CACS and obstructive CAD. This could be related to sample selection and study design differences. We included symptomatic patients suspected with CAD, while the previous studies evaluated asymptomatic individuals without any known cardiac disease. Therefore, we guess that higher NLR values may indicate active plaque formation. In patients with established plaques, increased anti-inflammatory responses might be the reason for diminished NLR values.

Relatively small sample size, retrospective design and lack of asymptomatic control group are important limitations to the present study which warrant further caution in interpreting the results. Additional prospective studies with larger sample sizes and more robust designs are needed.

## Conclusion

In conclusion, we found a direct association between higher CACS and obstructive patterns in coronary CT angiography. Our findings indicate that the possibility of the presence of obstructive CAD was higher among symptomatic patients with older age, lower NLR, and CACS>100.

## Competing interests

None declared.

## Acknowledgements

The authors acknowledge all of the participants of the study as well as the radiology department and of Rajaie cardiovascular medical and research center that supported this study for collecting data.

## Ethical approval

The protocol of this study was reviewed and approved by the Institutional Review Board and the Ethics Committee of Iran University of Medical Sciences (the ethical code No. IR.IUMS.FMD.REC1396.9411171011). An informed consent form was signed by all subjects prior to inclusion.

## Funding

None.
